# A new approach to broaden the range of eye colour identifiable by IrisPlex in DNA phenotyping

**DOI:** 10.1038/s41598-022-17208-w

**Published:** 2022-07-27

**Authors:** Ersilia Paparazzo, Anzor Gozalishvili, Vincenzo Lagani, Silvana Geracitano, Alessia Bauleo, Elena Falcone, Giuseppe Passarino, Alberto Montesanto

**Affiliations:** 1 Department of Biology, Ecology and Earth Sciences, University of Calabria, 87036 Rende, Italy; 2 Toptal, LLC, 2810 N. Church St. #36879, Wilmington, DE 19802-4447 USA; 3 Ivane Javakhishvili Tbilisi State University, 0162 Tbilisi, Georgia; 4 Institute of Chemical Biology, Ilia State University, 0162 Tbilisi, Georgia; 5 Biological and Environmental Sciences and Engineering Division (BESE), King Abdullah University of Science and Technology KAUST, Thuwal, 23952 Saudi Arabia; 6 BIOGENET, Medical and Forensic Genetics Laboratory, 87100 Cosenza, ASP Italy

**Keywords:** Genetic association study, Genetic markers

## Abstract

IrisPlex system represents the most popular model for eye colour prediction. Based on six polymorphisms this model provides very accurate predictions that strongly depend on the definition of eye colour phenotypes. The aim of the present study was to introduce a new approach to improve eye colour prediction using the well-validated IrisPlex system. A sample of 238 individuals from a Southern Italian population was collected and for each of them a high-resolution image of eye was obtained. By quantifying eye colour variation into CIELAB space several clustering algorithms were applied for eye colour classification. Predictions with the IrisPlex model were obtained using eye colour categories defined by both visual inspection and clustering algorithms. IrisPlex system predicted blue and brown eye colour with high accuracy while it was inefficient in the prediction of intermediate eye colour. Clustering-based eye colour resulted in a significantly increased accuracy of the model especially for brown eyes. Our results confirm the validity of the IrisPlex system for forensic purposes. Although the quantitative approach here proposed for eye colour definition slightly improves its prediction accuracy, further research is still required to improve the model particularly for the intermediate eye colour prediction.

## Introduction

Forensic DNA Phenotyping (FDP) is an emerging field of forensic genetics aimed at prediction of externally visible characteristics (EVC) of unknown sample donors directly from biological materials found at the crime scene. This approach is expected to provide clues helping investigators reduce/prioritize their list of suspects and make police investigations more rapid, efficient and less expensive^1–3^. While forensic genetic research is searching for additional phenotypic characteristics for predicting human appearance, those related to the pigmentations (eye, skin and hair colour) are today among the ones best characterized and validated^4^. In this context, eye colour is the best investigated phenotype for forensic genetic applications. In fact, a lot of genetic variants have been successfully identified in relation with iris pigmentation^5–9^. Some of these variants constitute the so-called IrisPlex system that to date represents the most popular model for eye colour prediction^10^. This system is based on the analysis of six Single Nucleotide Polymorphisms (SNP) located in six different genes: rs12913832 (*HERC2*), rs1800407 (*OCA2*), rs12896399 (*SLC24A4*), rs16891982 (*SLC45A2*), rs1393350 (*TYR*) and rs12203592 (*IRF4*). The IrisPlex model is based on a multinomial logistic regression model by which each individual is classified as being brown, blue or intermediate^10,11^. The parameters of such a model were initially estimated using phenotype and genotype data from 3804 Dutch individuals. In particular, genetic data are modelled in an additive fashion (number of minor alleles in the genotype) and the highest probability of all 3 categories was taken as the predicted iris colour of that individual. Using this model, very accurate prediction values were obtained for brown and blue eyes, while the prediction of intermediate colour is less precise. There have been several attempts to refine the IrisPlex system to improve its predictive value. These were based on both an increased number of analysed genetic variants and a different statistical modelling strategy^12–14^. However, despite these precautions, these alternative systems did not obtain the desired effects since recent data showed that the IrisPlex system still was the best performing model for eye colour prediction^15^. Eye colour is usually described qualitatively using subjective and visually defined phenotype categories. This discretization approach oversimplifies the quantitative nature of the trait causing an inevitably loss of information^2^. For this reason, several authors proposed quantitative measurements of iris colour^16–19^. This strategy not only allowed in the past years the identification of new genetic variants, but also the determination of a genetic model able to explain about 50% of quantitative eye colour variation^17^. Anyhow, the introduction of these measurements requires a methodology able to capture eye/hair colour in its fully continuous spectrum as accurately as possible^2^ since current models for eye colour prediction, such as the IrisPlex system, are not able to handle this kind of data.

The aim of this present study is to introduce a new quantitative approach for eye colour prediction using the well-validated IrisPlex system and high-resolution digital images and genotype data from 238 individuals from a Southern Italian population. To this purpose, several alternative iris colour categorizations were evaluated and inserted within the frame of the IrisPlex model for improving its classification accuracy.

## Results

Table 1 reports the minor allele frequencies for each SNP in the analysed sample together with the p-values of test of departure from Hardy–Weinberg equilibrium (HWE). All polymorphisms complied with HWE except rs12913832 located within the *HERC2* gene.

**Table 1:** Minor allele frequency (MAF) for each SNP, along with Hardy–Weinberg Equilibrium (HWE) p-value.

SNP	Alleles	MAF	HWE
HERC2-rs12913832	A/G	31.3	0.030
OCA2-rs1800407	G/A	4.9	1.000
SLC45A2-rs16891982	G/C	15.4	1.000
TYR-rs1393350	G/A	17.7	1.000
SLC24A4-rs12896399	G/T	26.6	0.423
IRF4-rs12203592	C/T	6.0	0.604

### Eye colour categorization

The visual inspection produced the following eye colour distribution in the analysed sample: 29 blue (3 blue-grey and 26 sky-blue), 55 intermediate (34 chestnut-green and 21 green), and 154 brown (52 light brown and 102 dark brown).

### Eye colour quantification using clustering algorithms

In order to obtain an objective eye colour classification, several clustering algorithms were applied on the CIELAB parameters. Table 2 reports the clustering solutions with the highest Silhouette index and four different clusters (see Supplementary Table 1 for the full list of explored clustering solutions).

**Table 2:** selection of solutions from the clustering analysis. For each solution,the respective clustering algorithm, whether the data were normalized or used in the original CIELAB values, the number of clusters, as well as the silhouette and adjusted Rand index value are reported. Full list in Supplementary Table 1.

Algorithm	Data preprocessing	Number of clusters	Silhouette value	Adjusted rand index
K-means	Original	4	0.407	0.332
BIRCH	Original	4	0.389	0.315
K-means	Normalized	4	0.372	0.381
K-medoids	Normalized	4	0.342	0.381
SC	Normalized	4	0.309	0.396

**Figure 1:**
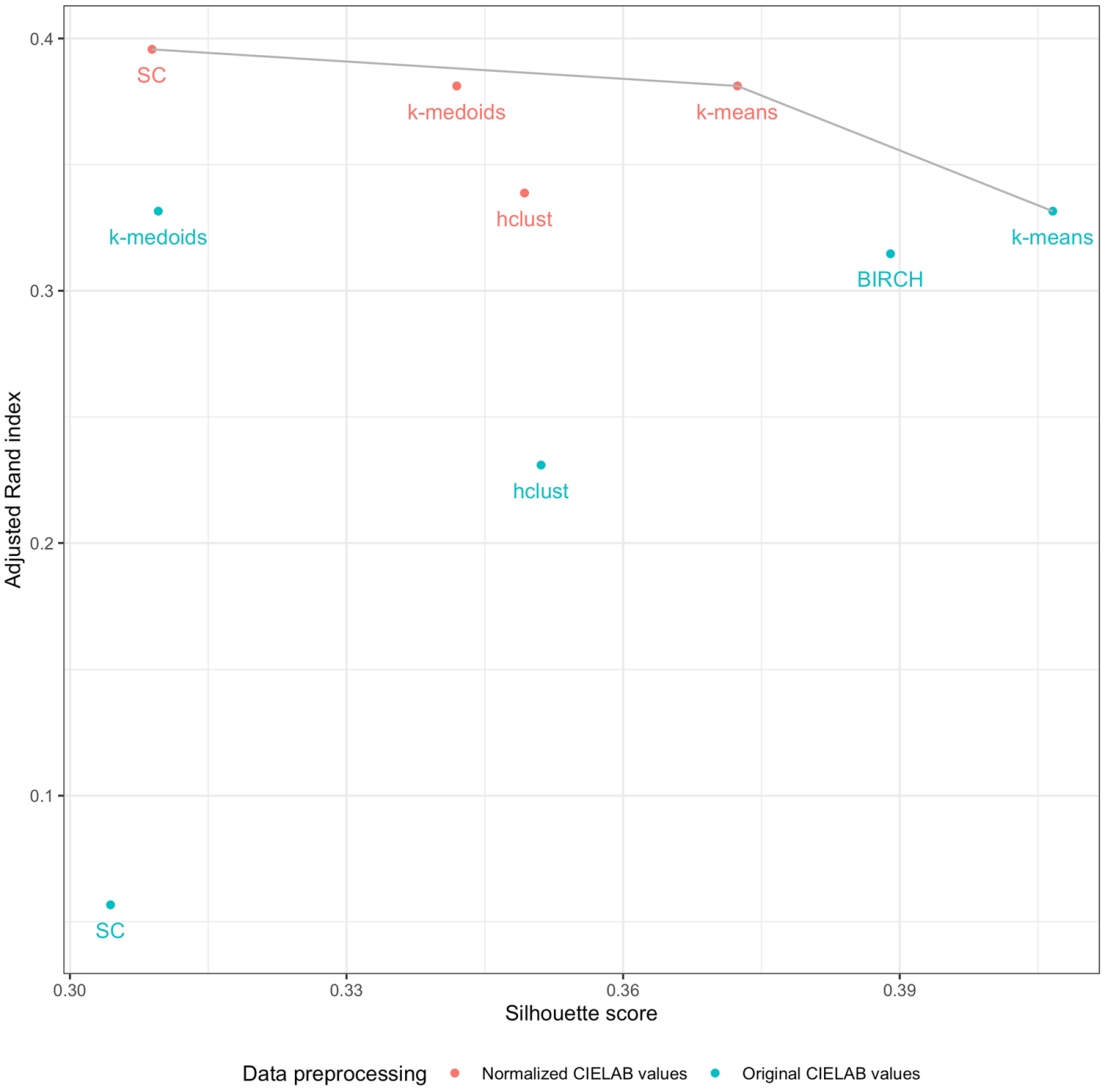
clustering performances. Each point represents a clustering solution, with the x-axis reporting the corresponding silhouette score, the y-axis the adjusted Rand index, and the colour indicating whether the CIELAB values were normalized. The Pareto front is represented as a grey line connecting the Pareto-optimal solutions (k-means solutions as well as SC with original CIELAB values).

We select the best clustering model based on a Pareto-optimal criterion; solutions that were top-ranked in either silhouette or adjusted rand index were deemed the optimal ones (see Fig. 1). According to this criterion, k-means with both original and normalized data, and SC with normalized data were chosen for subsequent analyses.

**Figure 2:**
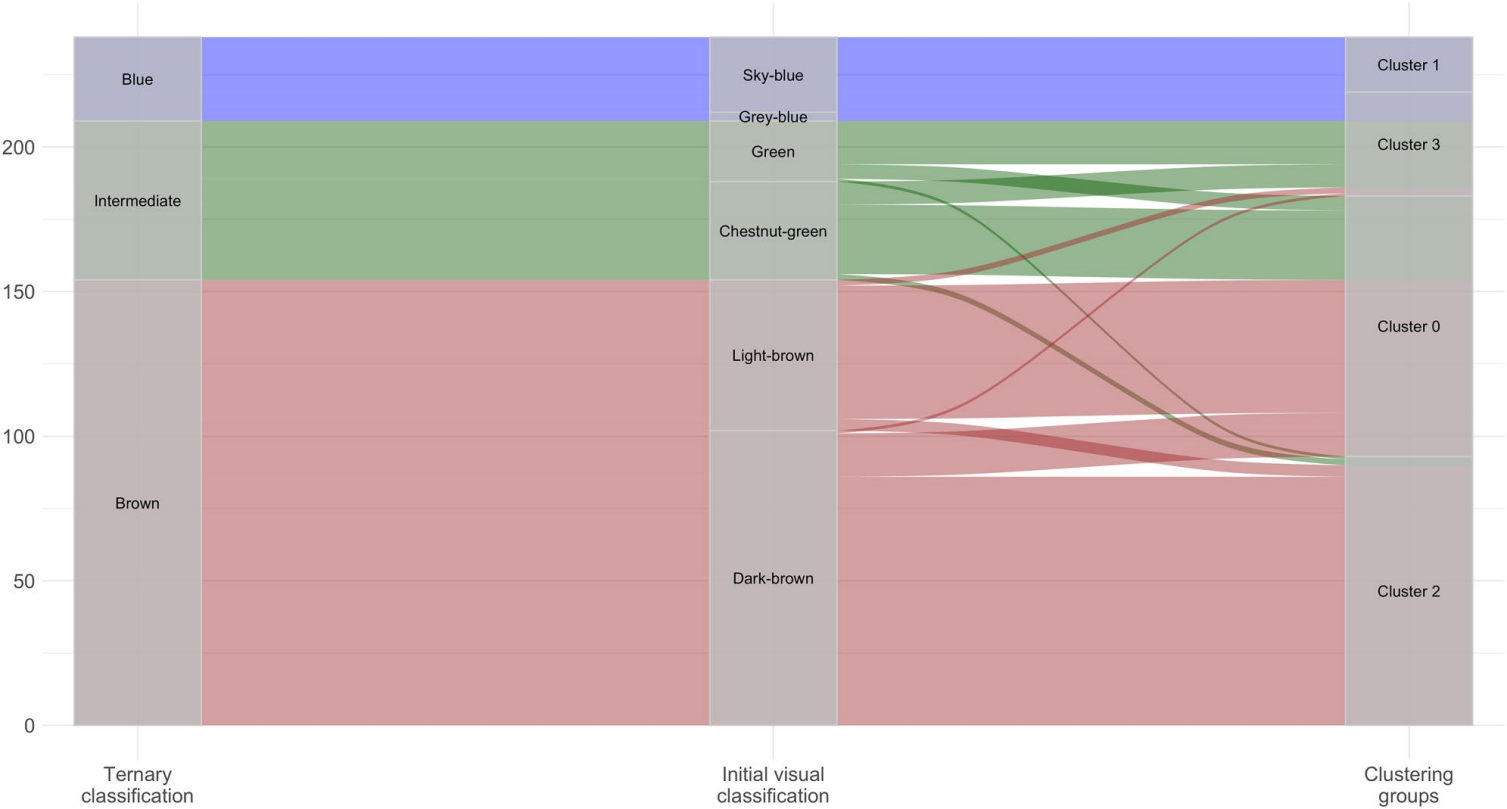
Alluvial plot showing sample distributions across different classifications. From left to right: ternary classification as used in the IrisPlex system, initial visual classification, and clustering groups as defined by k-means applied on the original CIELAB values.

Alluvial plots (Fig. 2, Supplementary Figs. 1 and 2) show the distribution of the three-category classification of the IrisPlex model (blue, intermediate and brown) across a more detailed initial visual classification (sky-blue, grey-blue, green, chestnut-green, light-brown and dark-brown) and the groups produced by the selected clustering algorithms.

We then labelled each cluster according to the prevalence of the colour flows into the cluster itself. For all clustering solutions, cluster 1 was labelled as blue, cluster 3 as intermediate and both cluster 0 and 2 as brown. In general, all clustering results allowed to distinguish between a light and a dark intermediate colour (cluster 3 and cluster 0, respectively).

### Contrasting IrisPlex predictions against eye-colour labels obtained by visual inspection and clustering analysis

Table 3 reports the overall accuracy obtained by the IrisPlex model on our cohort, according to different levels of thresholding and different eye colour definition. IrisPlex performances generally improve with higher threshold values. Most relevantly, it is clearly visible that the overall accuracy increases when the eye-colour labels defined by the k-means clustering algorithm are considered, with the original CIELAB values (not normalized) giving the best results.

**Table 3:** IrisPlex accuracy obtained for different levels of thresholding (rows) and different eye colour definition (columns).

Threshold	Visual classification	K-means (not normalized)	K-means (normalized)	SC (normalized)
0.7	0.795	0.865	0.851	0.763
0.5	0.765	0.848	0.835	0.730
0	0.744	0.832	0.811	0.706

Since the best-performing clustering solution was the k-means on the original (non-normalized) data, all subsequent analyses were performed based on eye-colour defined on the basis of such an algorithm.

Figure 1 shows the number of correct, incorrect, and undefined predictions at each threshold value and for (a) the eye-colour defined by visual inspection, (b) eye-colour defined through k-means clustering on the original (non-normalized) CIELAB values. The histograms indicate that applying a threshold improves the overall performance of the model because mostly incorrect predictions are turned into inconclusive ones. In other words, low confidence predictions are most likely incorrect, and excluding them from the evaluation increases the overall model performance.

In order to investigate this increase in accuracy, Fig. 4 dissects the model predictions according to eye colour and classification threshold. The eye-colour classification obtained by the clustering analysis provided performances in terms of accuracy higher than those obtained using eye-colour classification by visual inspection. In particular, the clustering analysis reclassified as brown a substantial number (29) of samples labelled as intermediate by the visual inspection, and this reclassification agrees with the IrisPlex which classifies these same samples as brown as well. Notably, the clustering analysis operates exclusively on the CIELAB values, while the IrisPlex solely analyses the genomic data, thus these two independent sources of information agree on this reclassification.

Regarding the effect of thresholding, it can be observed that increasing the threshold to 0.7 redefined as undefined the brown eyes that are incorrectly predicted as blue. Brown eyes became inconclusive by 3.2% (5 out of 154) for eye colour defined by visual inspection and 7.1% (13 out of 183) for the clustering-based approach, respectively. Blue eyes predicted as brown were reduced by 40% (2 out of 5) for eye colour defined by visual inspection and 66.7% (2 out of 3) for the clustering approach. Intermediate eyes were never predicted as intermediate but incorrect predictions as brown decreased by 26.9% (14 out of 52) for eye-colour defined by visual inspection; for eye-colour defined by clustering analysis, applying a 0.7 threshold reduces the number of incorrect predictions by 22.2% (8 out of 36 samples becomes undefined).

**Figure 3:**
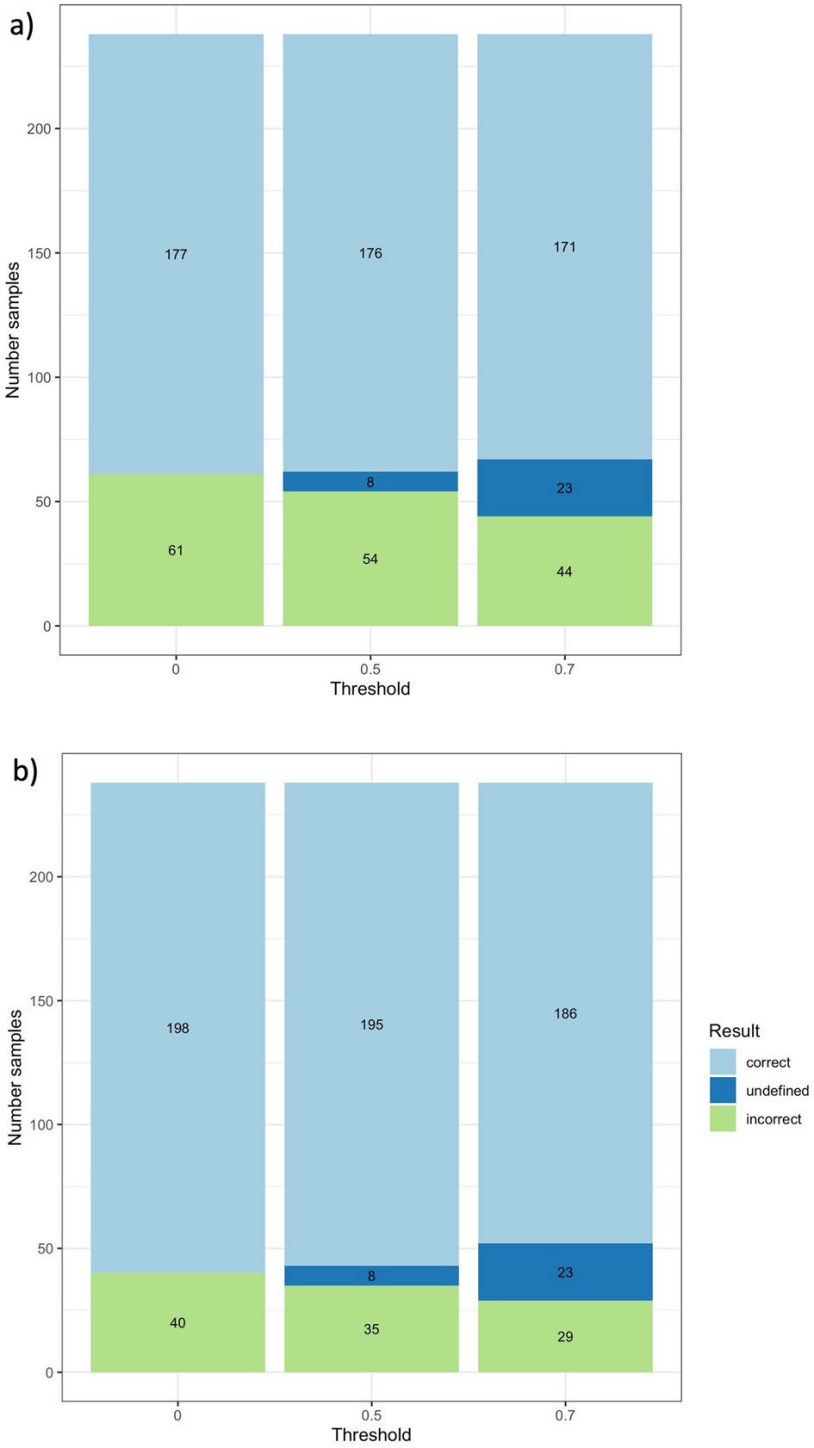
IrisPlex results. Each panel shows the number of correct, incorrect and undefined predictions at each threshold value. Particularly, panel (**a**) shows the results obtained with the eye-colour defined by visual inspection, and panel (**b**) with the eye-colour defined through k-means clustering on the original (non-normalized) CIELAB values.

**Figure 4:**
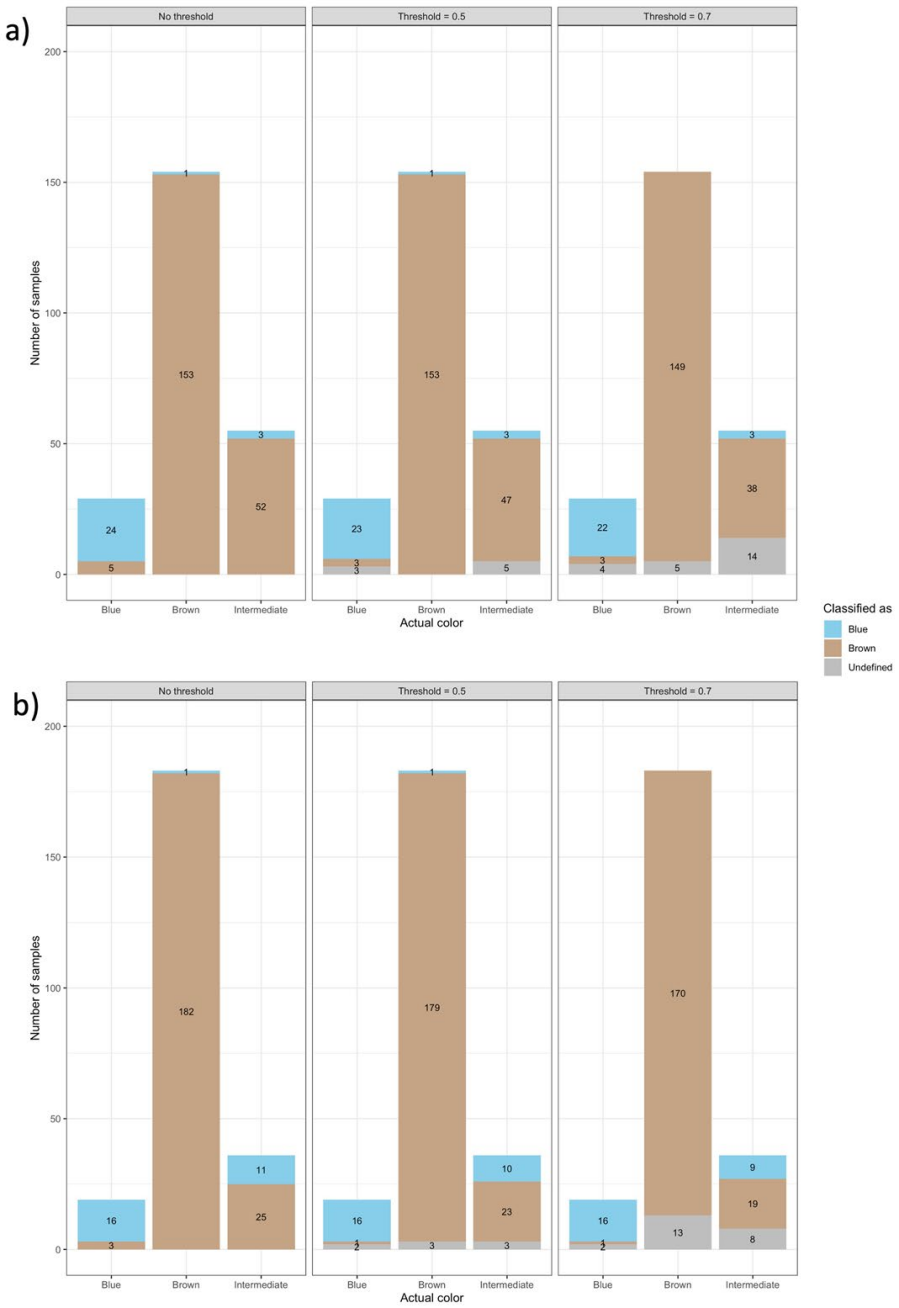
IrisPlex results dissected by eye-colour and threshold. Each panel shows blue, brown and intermediate eyes are classified. Panel (**a**) shows the results obtained with the eye-colour defined by visual inspection, and panel (**b**) with the eye-colour defined through k-means clustering on the original (non-normalized) CIELAB values.

In Table 4 the classification metrics for each colour category and threshold value are reported, both for the eye-colour defined by visual inspection and clustering analysis. It is clearly visible that all the performance metrics were improved by applying a threshold, as shown also in Figs. 3 and 4. Using the eye classification provided by the clustering analysis clearly improves the specificity for the brown category, mainly due to reclassification as brown of several intermediate samples recognized as brown also by the IrisPlex model. We also observe a decrease in the specificity and PPV for the blue colour, due to the reclassification of 8 samples from blue (visual inspection) to intermediate and classified as blue by IrisPlex.

**Table 4:** Detailed performance metrics by eye colour and threshold. Results shown both for eye-colour defined through visual inspection and clustering algorithms.

Colour	Threshold	Visual inspection eye-colour					Clustering-based eye-colour (k-means on non-normalized data)				
		ACC	Sensitivity	Specificity	PPV	NPV	ACC	Sensitivity	Specificity	PPV	NPV
Blue	0	0.962	0.828	0.981	0.857	0.976	0.937	0.842	0.945	0.571	0.986
Blue	0.5	0.970	0.885	0.980	0.852	0.985	0.948	0.941	0.948	0.593	0.995
Blue	0.7	0.972	0.880	0.984	0.880	0.984	0.953	0.941	0.955	0.640	0.995
Brown	0	0.756	0.994	0.321	0.729	0.964	0.878	0.995	0.491	0.867	0.964
Brown	0.5	0.778	0.994	0.342	0.754	0.963	0.891	0.994	0.520	0.882	0.963
Brown	0.7	0.809	1.000	0.379	0.784	1.000	0.907	1.000	0.556	0.895	1.000
Intermediate	0	0.000	0.000	1.000	0.000	0.769	0.000	0.000	1.000	0.000	0.849
Intermediate	0.5	0.000	0.000	1.000	0.000	0.783	0.000	0.000	1.000	0.000	0.857
Intermediate	0.7	0.000	0.000	1.000	0.000	0.809	0.000	0.000	1.000	0.000	0.870

**Figure 5:**
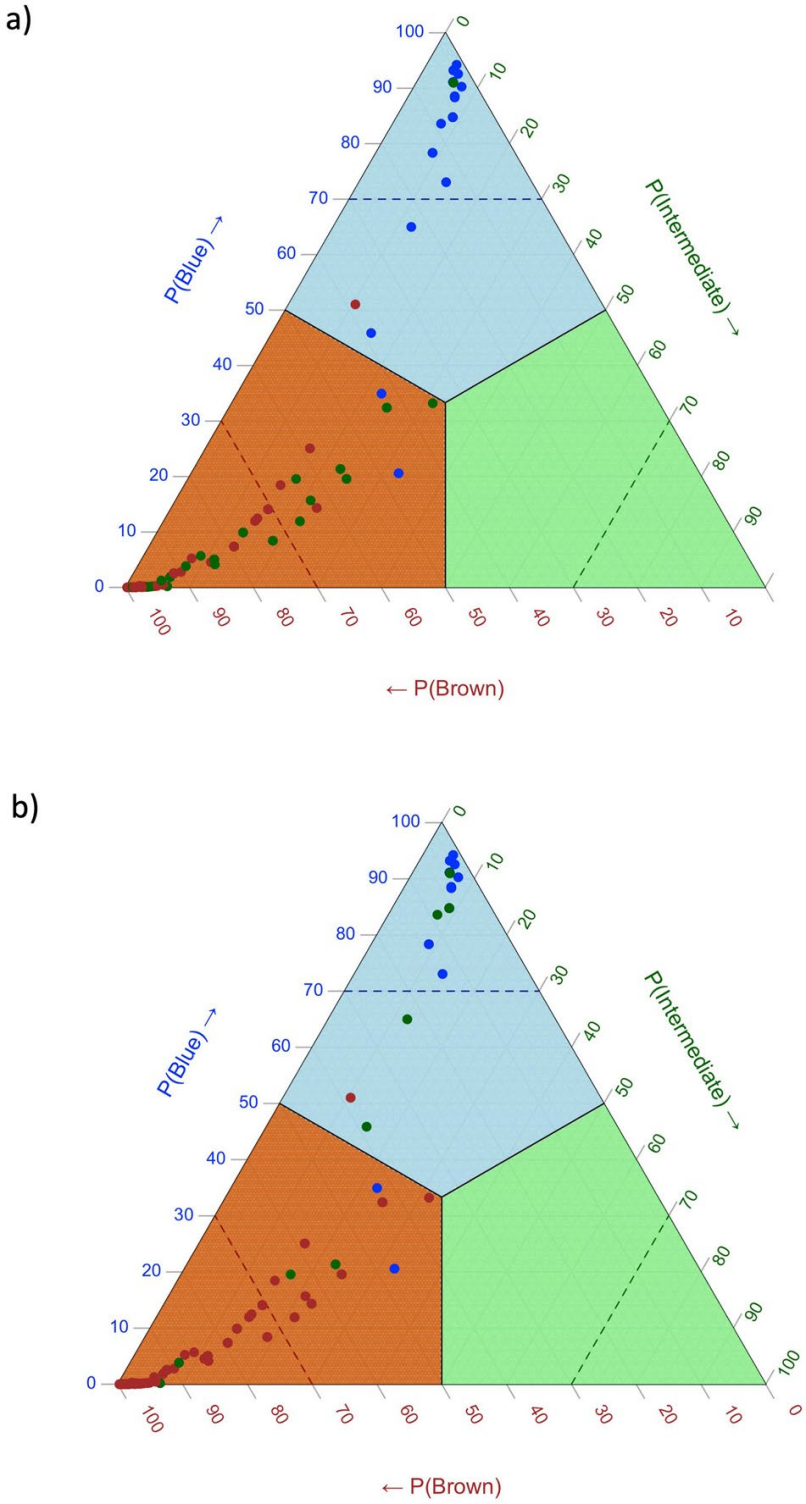
Ternary plots representing, for all samples (dots), the probabilities provided by the IrisPlex system. Panel (**a**) shows the results obtained with the eye-colour defined by visual inspection, and panel (**b**) with the eye-colour defined through k-means clustering on the original (non-normalized) CIELAB values.

The ternary plots in Fig. 5 show the probabilities produced by the IrisPlex system. We highlight the IrisPlex model difficulties in separating the intermediate category from brown. Basically, no threshold can well separate brown and intermediate examples. Intermediate samples fell in both blue and brown sector almost equally when the eye colour was defined through clustering analysis (panel b), while they were mostly concentrated in the brown section in the case of the labels defined by visual inspection.

These plots also clearly underline the samples deemed as intermediate by visual inspection that become brown according to the clustering analysis (points switching from green to brown in the bottom left corner of the panel b) as well as the blue samples (visual inspection) turning into intermediate (clustering analysis) in the top corner of the two triangles.

## Discussion

In the present study the efficacy of the IrisPlex model for eye colour prediction was analyzed in 238 individuals of Italian ancestry to evaluate their possible applicability as a tool of DNA intelligence in forensic investigations. Our results confirm the previous findings from several different populations showing once again that the IrisPlex system predicts blue and brown eye colour with high accuracy while it is inefficient in the prediction of intermediate eye colour^20–25^. Indeed, the accuracy values for blue and brown eye colour categories in our sample were very high and equal to 0.972 and 0.809, respectively, while no one intermediate eye colour was correctly predicted as previously reported in another Italian sample^15^.

Here, we quantified continuous eye colour variation into CIELAB colour space using high-resolution digital full-eye photographs following the procedure reported in Edwars^19^. Clustering algorithms applied on the CIELAB parameters allowed us to obtain a standardized and objective measurement of eye colour, as well as, a better and more precise definition of the phenotype under study. Slightly improved results were obtained when this clustering-based approach was used for eye colour classification. In particular, using several clustering algorithms applied on quantitative measurements of iris colour, we obtained an improved classification performance especially for the clustering-based brown category.

The clustering-based approach here proposed, likewise other similar quantitative approaches for eye colour definition, may also be exploited as a standardized and objective measurement of eye colour useful also because it makes possible to directly compare results from different studies. In fact, one of the most important limitations affecting the development of a genetic model for eye colour prediction is the definition of the phenotype. Subjective interpretations of eye colour, by oversimplifying the quantitative nature of the trait and causing an inevitably loss of information, makes it difficult to compare and validate the results obtained in different populations and this also affects the classification performance of the adopted model.

There have been several attempts to refine the IrisPlex system to improve its predictive value mainly focused on the increase in the number of genetic variants^12–14^. This approach did not obtain the desired effects since the IrisPlex system still represents the best performing model for eye colour prediction. Within this context, another very promising approach seems to be the inclusion of epigenetic markers. In fact, several authors observed that the hect domain and RCC1-like domain 2 (*HERC2*) rs12913832 variation, the marker of the IrisPlex system with the highest discrimination power, is located in an enhancer element that regulates the expression of *OCA2* gene^7^. In addition, it was also shown that *OCA2* expression was reduced in lightly pigmented melanocytes with the rs12913832-G variant with respect to darkly pigmented melanocytes with the A allele^7,26^. In agreement with this observation, the inclusion of epigenetic markers in the IrisPlex model might be useful to improve its prediction accuracy and in particular for the non-blue and non-brown eye colours.

The aim of this work was to test the predictive capabilities of the IrisPlex system, using eye colour definitions based both on visual inspection and on quantitative approach (clustering). Consequently, we based our attention to the clustering solutions in which three or more groups were identified, discarding clustering solutions identifying only two eye colours, since testing the IrisPlex predictions on these solutions would have been problematic. However, an interesting study carried out by Meyer et al. clearly showed that the perception of intermediate eye colour varies greatly among individuals, and this represents the main reason why using only two categories of eye colour (blue and brown) provides better results than a three-category system (blue, intermediate, and brown)^23^. In line with these results, the Section of Forensic Genetics in Denmark recently began offering eye colour prediction to the police using two categories of eye colour (blue and brown) through the analysis of rs12913832 variability. All these lines of evidence, together with our results, suggest that the current definition of eye colour based on visual inspection should either be re-defined on the basis of more quantitative criteria or should be dropped all together in favour or a two-colour definition.Although the quantitative approach here proposed for eye colour definition improves the prediction accuracy of IrisPlex system, further research is still required to improve the model performance particularly for the non-blue and non-brown eye colour prediction.

## Methods

### Sample

The present study was carried out at the Department Biology, Ecology and Earth Sciences of the University of Calabria within a recruitment campaign focused on students and staff of the University between November 2018 and October 2019. 238 individuals (72 men and 166 women) were recruited. Trained staff members administered a brief and standardized questionnaire in order to obtain information regarding the socio-demographic data. During the interview, eye images using a professional camera were obtained and buccal swabs were collected as source of DNA. Written informed consent was obtained from all recruited individuals. The study was approved by the Ethics Committee of University of Calabria (Prot. NP-5942018) and met the criteria of the Helsinki declaration.

### Digital photographs

Photographs were taken at a distance of approximately 10 cm of each individual’s left iris under similar light conditions with a Nikon P300 with 100 mm f/1.8 NIKKOR Optical Zoom Lens, ISO 800. A coaxial biometric illuminator was used to deliver a constant and uniform source of light to each iris at 5,500 K (D55 illuminant).

### Classification of eye colour by visual inspection of digital photographs

Iris colour was classified qualitatively by human visual identification as already described in other studies^15,20,21,25^. Briefly, each eye image was graded independently by 2 different observers who classified eye colours into four categories: blue (including blue-grey and sky-blue), green (including green, and green with brown iris ring), chestnut-green (including peripheral green central brown, brown with some peripheral green) and brown (including light brown and dark brown). In order to keep the three-category classification of the IrisPlex model and to ensure consistency across studies, we mapped green and chestnut-green categories to intermediate category. Note that these two categories correspond to light intermediate and dark intermediate classes described in other studies^15,22,25^. A third observer was consulted to resolve inconsistencies through majority-voting and to assess the final eye colour of each volunteer. Overall, 91% (217/238) of the classifications showed complete agreement between the 2 observers. Of the 21 remaining discrepancies, 18 were between light brown and chestnut-green, finally classified 17 as light brown, one as chestnut-green; the remaining discrepancy was between sky-blue and green, finally classified as green.

### Quantitative eye colour

Image processing was based on the procedure reported in Edwards and colleagues using the dedicated webtool^19^. In brief, after the scleral, pupillary and collarette boundaries are defined, the application automatically extracts a measurement of average eye colour starting from a 60° angle wedge taken from the left side of the iris. The web application also isolates the portion of the wedge that represents the ciliary zone and the portion of the wedge that represents the pupillary zone. At the end of this procedure, for each iris image, the average RGB value of the entire wedge, the ciliary and the pupillary zones are obtained. The obtained RGB values are then converted into in CIE 1976 L*a*b* (CIELAB) colour space. In this colour space, the L* coordinate represents the lightness dimension and ranges from 0 to 100, with 0 being black and 100 being white. The red/green colours are represented along the a* coordinate, with green at negative a* values and red at positive a* values. The yellow/blue colours are represented along the b* coordinate, with blue at negative b* values and yellow at positive b* values.

Although several automated methods have been developed to facilitate the isolation of the iris from photographs of the eye^17,18,25^, the method here adopted as reported in Edwards et al^19^, appears to be superior as it allows to manually define the boundaries of the iris and to separate the eye into different regions. Since the left quadrant of the iris was least likely to be obstructed by eyelashes and eyelids, it would bias the colour of the iris towards the pupillary region, we selected a wedge to represent iris colour instead of the entire iris.

### Classification of eye colour using an unsupervised machine learning approach

In order to make eye colour categorization process more objective, a cluster analysis approach based on the coordinates in CIELAB space was carried out. To this purpose, several clustering algorithms were experimented, including Affinity Propagation (AP)^27^, Balanced Iterative Reducing and Clustering using Hierarchies (BIRCH)^28^, Density-Based Spatial Clustering of Applications with Noise (DBSCAN)^29^, hierarchical clustering (hclust)^30^, k-means^31^, k-medoids^32^, k-modes^33^, mean-shift^34^, Ordering Points To Identify the Clustering Structure (OPTICS)^35^, and Spectral Clustering (SC)^36^. The settings adopted for each of the algorithms is indicated in Supplementary Table 1. Each clustering algorithm was applied on the original CIELAB values as well as on normalized values. The Euclidean distance was used in conjunction with all the methods requiring a distance metric. Preliminary analyses with a distance metric specifically designed for the CIELAB space, namely the CIEDE2000^37^, produced results comparable with the ones obtained with the Euclidian distance. Thus, we decided to only use the latter, simpler metric rather than CIEDE2000. The optimal clustering solution was chosen according to the silhouette criterion^38^, while the agreement of each clustering solution with the categorization obtained through visual inspection was assessed through the adjusted rand index^39^. It should be noticed that among the clustering solutions identified by cluster analysis, since the IrisPlex model was developed for the prediction of three eye colour categories, we evaluated only the solutions providing at least three groups. In particular, solutions with four groups were taken into account only because we condensed two clusters in a single intermediate category.

### Genetic markers

Genetic profiling was carried out on the DNA extracted from buccal swab samples by analysing the genetic polymorphisms included in the IrisPlex^10^. Genotyping was performed using TaqMan genotyping assays following manufacture’s instruction and 10 ng of DNA mixed with the TaqMan Genotyping Master Mix (Thermo Fisher Scientific).

### The IrisPlex model

From a statistical point of view the IrisPlex system exploits a multinomial logistic regression model by which each individual is classified as being brown, blue or intermediate based on the three obtained prediction probabilities^10^. The parameters of such a model were estimated using phenotype and genotype data modeled in an additive fashion (number of minor alleles in the genotype). Prediction with the IrisPlex model were obtained using the dedicated webtool (https://hirisplex.erasmusmc.nl/). As suggested by the authors, the predicted colour was the one with a probability higher than the threshold of 0.7. Individuals with all the colour probabilities under 0.7 were marked as “undefined”. Additionally, we also applied a threshold of 0.5. When no threshold was applied, the predictions were assigned to the colour with the absolute highest probability. In this last case, individuals that obtained equal probabilities for multiple (two or three) colour categories were classified as intermediate.

## Supplementary Information

Supplementary Information.

## Data Availability

The dataset generated during and/or analysed during the current study are not publicly available due to ethical concerns but is available from the corresponding author on reasonable request.
