# Supplementary material for: A new approach to broaden the range of eye colour identifiable by IrisPlex in DNA phenotyping
Source: Sci Rep. 2022 Jul 27;12:12803. doi: 10.1038/s41598-022-17208-w (PMC9329466; doi:10.1038/s41598-022-17208-w)
Supplement: Supplementary file 1 — Supplementary Information. [file 41598_2022_17208_MOESM1_ESM.docx]

**Supplementary Materials**

Supplementary Tables

Supplementary Table 1: complete clustering results. For each solution we report the respective clustering algorithm, whether the data were normalized or used in the original CIELAB values, the number of clusters, as well as the silhouette and adjusted Rand index value.

| Algorithm | Data Preprocessing | Number of Clusters | Silhouette Value | Adjusted Rand Index |
| --- | --- | --- | --- | --- |
| Mean Shift | normalized data | 2 | 0.52770001 | 0.5633894 |
| Mean Shift | original data | 2 | 0.48910001 | 0.48546671 |
| K-means | original data | 4 | 0.4066 | 0.33154957 |
| BIRCH | original data | 4 | 0.389 | 0.31465603 |
| K-means | normalized data | 4 | 0.37239999 | 0.3811878 |
| Hierarchical Clustering | original data | 4 | 0.3511 | 0.23090465 |
| Hierarchical Clustering | normalized data | 4 | 0.3493 | 0.33875248 |
| K-medoids | normalized data | 4 | 0.34195504 | 0.3811878 |
| K-medoids | original data | 4 | 0.30958319 | 0.33154957 |
| AP | original data | 17 | 0.30899999 | 0.09132933 |
| SC | normalized data | 4 | 0.3089 | 0.39571553 |
| SC | original data | 4 | 0.3044 | 0.05672896 |
| AP | normalized data | 15 | 0.29519999 | 0.12487027 |
| DBSCAN | original data | 1 | 0 | 0 |
| DBSCAN | normalized data | 1 | 0 | 0 |
| BIRTCH | normalized data | 1 | 0 | 0 |
| OPTICS | original data | 12 | -0.2278 | -0.0009027 |
| OPTICS | normalized data | 10 | -0.256 | 0.00462037 |
| K-modes | original data | 4 | -0.3124 | -0.0021754 |
| K-modes | normalized data | 4 | -0.3688 | -0.0021754 |

Supplementary Figures


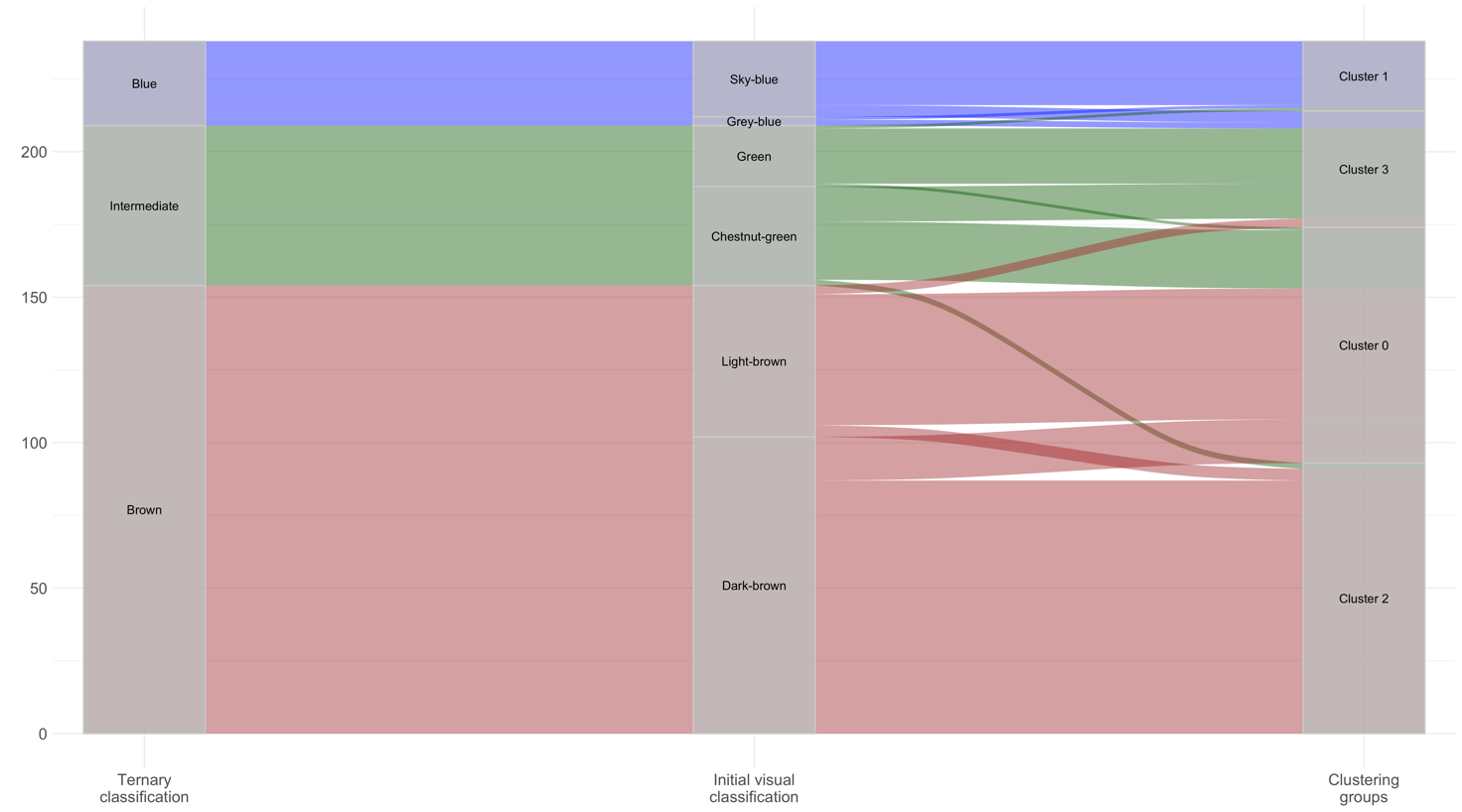


Supplementary Figure 1: alluvial plot showing sample distributions across different classifications. From left to right: ternary classification as used in the IrisPlex system, initial visual classification, and clustering groups as defined by k-means applied on the normalized CIELAB values.


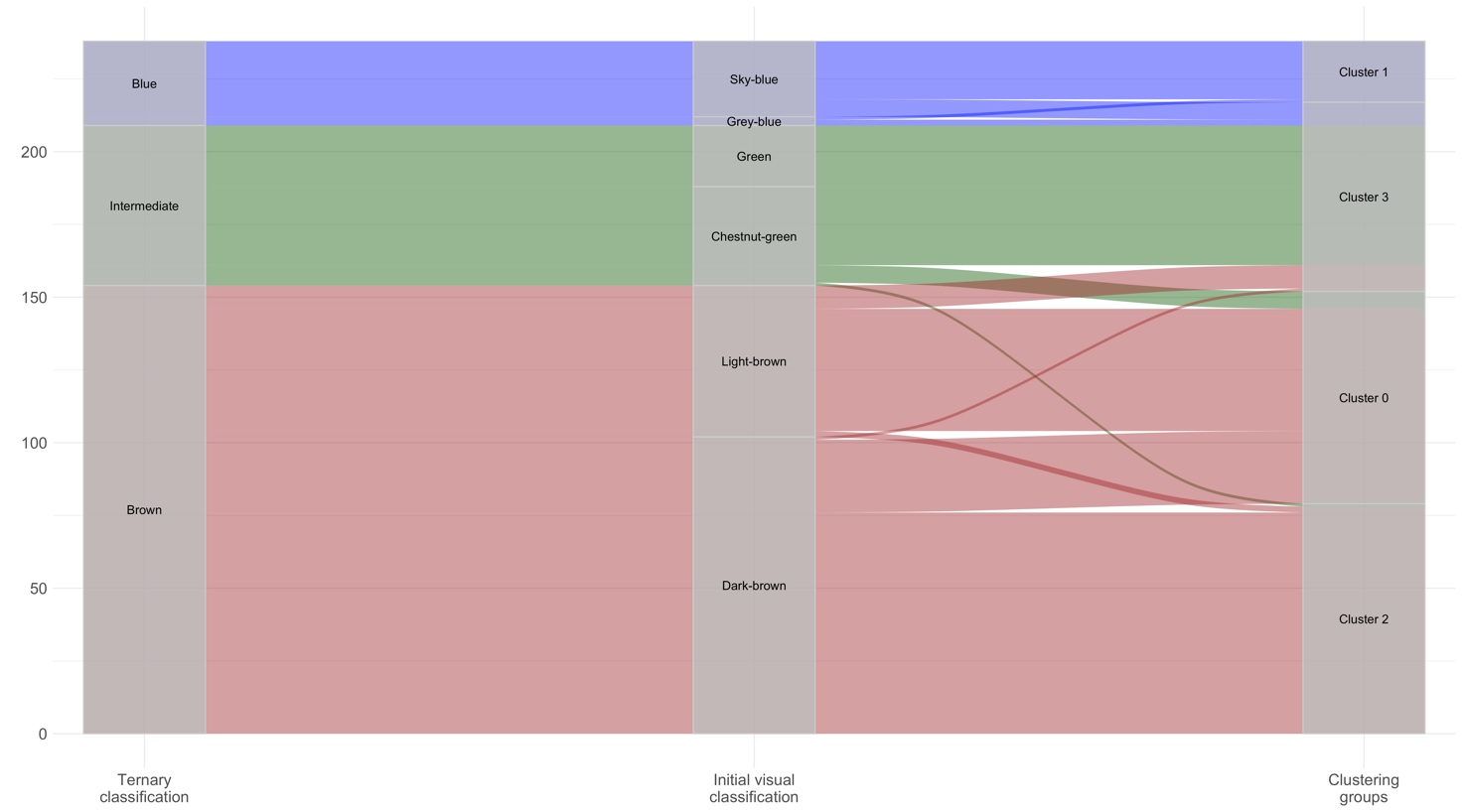


Supplementary Figure 2: alluvial plot showing sample distributions across different classifications. From left to right: ternary classification as used in the IrisPlex system, initial visual classification, and clustering groups as defined by SC applied on the normalized CIELAB values.
